# The Significance of the Intrinsically Disordered Regions for the Functions of the bHLH Transcription Factors

**DOI:** 10.3390/ijms20215306

**Published:** 2019-10-24

**Authors:** Aneta Tarczewska, Beata Greb-Markiewicz

**Affiliations:** Department of Biochemistry, Faculty of Chemistry, Wroclaw University of Science and Technology, Wybrzeże Wyspiańskiego 27, 50-370 Wroclaw, Poland; aneta.tarczewska@pwr.edu.pl

**Keywords:** bHLH, IDP, IDR, LLPS, disorder prediction, LLPS prediction, transcription, phase separation

## Abstract

The bHLH proteins are a family of eukaryotic transcription factors regulating expression of a wide range of genes involved in cell differentiation and development. They contain the Helix-Loop-Helix (HLH) domain, preceded by a stretch of basic residues, which are responsible for dimerization and binding to E-box sequences. In addition to the well-preserved DNA-binding bHLH domain, these proteins may contain various additional domains determining the specificity of performed transcriptional regulation. According to this, the family has been divided into distinct classes. Our aim was to emphasize the significance of existing disordered regions within the bHLH transcription factors for their functionality. Flexible, intrinsically disordered regions containing various motives and specific sequences allow for multiple interactions with transcription co-regulators. Also, based on in silico analysis and previous studies, we hypothesize that the bHLH proteins have a general ability to undergo spontaneous phase separation, forming or participating into liquid condensates which constitute functional centers involved in transcription regulation. We shortly introduce recent findings on the crucial role of the thermodynamically liquid-liquid driven phase separation in transcription regulation by disordered regions of regulatory proteins. We believe that further experimental studies should be performed in this field for better understanding of the mechanism of gene expression regulation (among others regarding oncogenes) by important and linked to many diseases the bHLH transcription factors.

## 1. Introduction

The bHLH (basic Helix-Loop-Helix) proteins are the important family of transcription factors (TFs) present in all eukaryotes: from yeasts [1,2] and fungi [3] to plants [4] and metazoans [5,6,7,8,9,10]. All family members contain the HLH domain responsible for dimerization [11]. This domain is usually preceded by a stretch of basic residues which enable DNA binding [12]. The bHLH TFs recognize tissue-specific enhancers containing E-box sequences which regulate expression of a wide range of genes involved in cell differentiation and development [13].

Currently, a few independent classification systems of the bHLH proteins exists: evolutionary classification based on the phylogenetic studies of the bHLH proteins, which classify the bHLH family members into six A-F classes [7,8,14], and a new one based on the complete amino acid sequence analyses, classifying the bHLH proteins into six clades without assumptions about gene function [15]. Contrary to the previous methods, natural method of classification proposed by Murre [12], which divides the bHLH proteins into seven classes, is based on the presence of additional domains, expression patterns and performed transcriptional function [10]. For purposes of clarity, some attempts to revise and systematize different classification systems were undertaken [16]. In this review we present classification of bHLH proteins according to Murre [12], with some short description of presented classes (Table 1). 

Both class I (known as E proteins) and class II of the bHLH TFs do not possess domains additional to the bHLH. Contrary to the class I which is expressed in many tissues, the class II proteins expression is tissue specific. Members of the class II are dimerization partners for the class I transcription factors. Class III comprises proteins possessing Leucine-zipper (LZ) motif in addition to the bHLH. Important members of the class III are proteins belonging to the Myc subfamily, which regulate oncogenic transformation, apoptosis, and cellular differentiation. To class IV belong MAD and MAX which can dimerize with MYC and regulate its activity. Also, MAD/MAX are able to create homo- and heterodimers with each other. Although these TFs do not possess transcription activation domain (TAD), MAD/MAX dimers can influence the transcription in a differentiated way. Class V contains transcriptional inhibitors ID1-3 which are not able to bind DNA and act by the other bHLH proteins sequestration. Interestingly, the fourth member of this class- ID4 function as inhibitor of ID1-3 [42]. Class VI comprise proteins containing additional Orange domain adjacent C-terminally to the bHLH domain (bHLH-O). Transcription factors from the described classes perform regulatory function in various developmental processes including cells differentiation and maintaining pluripotency. For this reason they are often linked to cancer development. Class VII comprise transcription factors which possess PAS (Period-Aryl hydrocarbon receptor nuclear translocator-Single minded) domain located C-terminally to the bHLH domain. PAS domain is crucial for the bHLH-PAS proteins specifity [43]. Structurally, the C-terminal PAS domain is often associated with PAC (C-terminal to PAS) motif [44,45]. bHLH-PAS transcription factors are responsible for sensing environmental signals like the presence of xenobiotics (AHR), hypoxia (HIF) or setting of circadian rhythms of organism (CLOCK, CYCLE, BMAL). The members of subclass II of bHLH-PAS TFs -ARNT proteins are general dimerization partners of the subclass I members.

## 2. The Role of the bHLH Proteins in Transcription

The regulation of genes expression by multiple transcription factors, cofactors and chromatin regulators establish and maintains a specific state of a cell. Inaccurate regulation of transmitted signals can results in diseases and severe disorders [46]. Therefore, transcription requires balanced orchestration of adjustable complexes of proteins. A key regulator of transcription is Mediator, a multi-subunit Mediator complex which interacts with RNA polymerase II (Pol II), and coordinates the action of numerous co-activators and co-repressors [47,48,49,50]. Function of the Mediator is conserved in all eukaryotes, though, the individual subunits have diverged considerably in some organisms [51,52].

Up to date, for some bHLH family representatives, interactions with subunits of the Mediator and/or chromatin remodeling histone acetyltransferases/deacyltransferase, were reported. In plants, the Mediator complex is a core element of transcription regulation important for their immunity [53]. It was shown, that in *Arabidopsis thaliana* important jasmonate signaling and resistance to fungus *Botrytis cinerea*, is dependent on the interaction between MED25 subunit of the Mediator and MYC2 [54,55,56], and interaction of MED8 subunit of the Mediator with FAMA belonging to the bHLH family [57]. Sterol regulatory element binding proteins (SREBPs) the class II bHLH TFs (Table 1) are transcription activators critical for regulation of cholesterol and fatty acid homeostasis in animals. It was shown that human SREBPs bind CBP/p300 acetyltransferase [58] and MED15 subunit of the Mediator to activate target genes [59]. Also yeast Ino2 was shown to bind MED15 subunit of the Mediator tail [60].

The representative of class II TFs TAL1 (Table 1) is required for the specification of the blood lineage and maturation of several hematopoietic cells. TAL1/SCL is considered as a master TF delineating the cell fate and the identity of progenitor and normal hematopoietic stem cells (HSCs). It regulates other hematopoietic TFs thus has a potential for cell reprogramming [22]. TAL1 also binds CBP/p300 acetyltransferase [61,62]. Similarly MyoD—a myogenic regulatory factor which controls skeletal muscle development binds CBP and recruits histone acetyltransferase to activate myogenic program [63]. Cao et al. showed that of MyoD modify the myoblasts chromatin structure and accessibility [64]. ASCL1 (class II, Table 1) was shown to be a pioneer factor which promotes chromatin accessibility and enables chromatin binding by others TFs [65]. Recently, also AHR (bHLH-PAS, Table 1) was suggested to be a pioneer factor which regulates DNA methylation during embryonic developments in unknown way [66]. In clear cell renal cell carcinoma (ccRCC), the most frequent mutation causes the von Hippel-Lindau (VHL) tumor suppressor inactivation leading to genome-wide enhancer and super-enhancer remodeling. This process is mediated by the interaction of HIF2α and HIF1β (bHLH-PAS, Table 1) with histone acetyltransferase p300 [67]. CLOCK, the other bHLH-PAS subfamily member (Table 1) was shown to mediate histone acetylation in a circadian time-specific manner [68].

Interestingly, the bHLH-O proteins members (class VI, Table 1) HEY proteins can function as transcription repressors as well as transcription activators. They were shown to bind directly DNA and interact with histone deacetylases and other TFs [28,69]. On the other hand, gene activation by HEY is regulated in an indirect way. Multiple HEY binding sites located downstream and close to the transcriptional start site, resulted in a hypothesis that HEY influence the pausing/elongation switch of Pol II [70]. Interestingly, though most of TFs stimulate transcription initiation, MYC (class III, Table 1) was shown to stimulate transcription elongation by recruitment of the elongation factor [71]. The presented studies indicate that the crucial role of the bHLH proteins in maintaining transcriptional regulation of important developmental (e.g., cell differentiation) and oncogenic pathways is dependent on the multiple interactions with basal transcriptional machinery.

## 3. The bHLH Transcription Factors as IDPs

Intrinsically disordered proteins (IDPs) discovered in 1990s obliterate the paradigm derived from Anfinsen’s work, stating that functional proteins must possess a well-defined, ordered, three dimensional structure [72]. Currently it is known, that a large number of proteins is perfectly functional or even multifunctional in a disordered state in which a polypeptide chain undergoes rapid conformational fluctuations [73,74,75,76]. Intrinsic disorder can be spread throughout the whole polypeptide chain, or it can be limited to intrinsically disordered regions (IDRs) of various length, which are accompanied by well folded domains [77]. The unique properties of disordered proteins originate from their unusual amino acids composition [78]. IDPs/IDRs are depleted in order promoting amino acid residues (hydrophobic, aromatic, aliphatic side chains). In contrast, they possess unusually high content of charged and hydrophilic amino acid residues [79,80,81]. As a consequence, disordered polypeptide chains have extremely high net charge and low hydrophobicity [82]. IDPs are pliable and highly dynamic molecules of interconvertible conformations. They may completely or almost completely lack the regular secondary structures. However, the content of secondary structure may also be quite significant and molecules can exist in a molten globule state [83,84,85]. Various in silico analyses indicated that the proportion of disordered proteins is drastically higher in eukaryotes comparing to prokaryotes [86]. This disproportion reflect the complexity of signaling pathways in which IDPs/IDRs play a crucial role [87]. Due to the flexible and dynamic nature, IDPs/IDRs can form fuzzy complexes, adopting various conformations [88]. According to this, one IDP can form multiple interactions with various partners. Due to a large accessibility of particular residues in a disordered chain, the interaction pattern can be easily modified by posttranslational modifications [89]. For that reason IDPs/IDRs often serve as molecular hubs, modulators and sensors of cellular signals [85].

**Figure 1:**
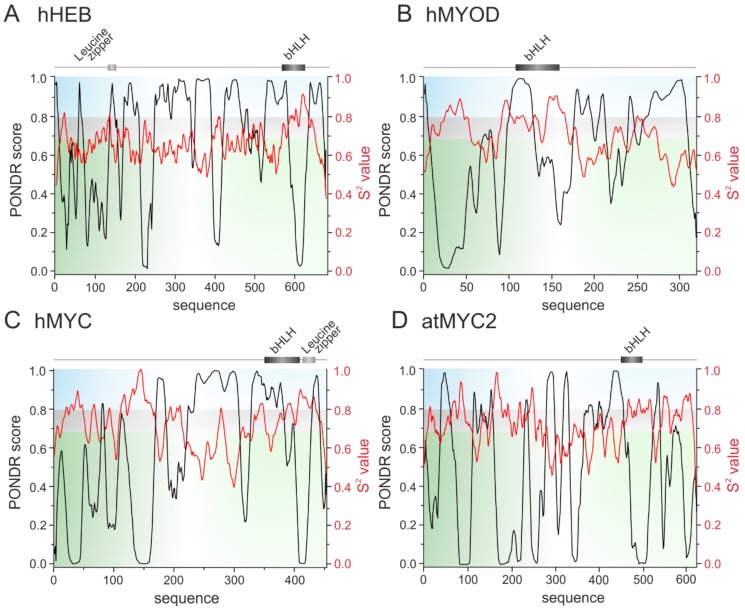
Prediction of intrinsically disordered regions. The top panel presents the domain structure of the analyzed bHLH proteins. Dark grey rectangle indicates the position of bHLH domain, the light grey Leucine zipper. The bottom panel presents a prediction of intrinsically disordered and flexible regions based on the amino acid sequence of proteins. Prediction were performed using PONDR-VLXT (left Y axis) and DynaMine (right Y axis) software. For PONDR prediction, a score above 0.5 indicates disorder. For DynaMine, a S^2^ value above 0.8 (blue zone) indicates rigid conformation, 0.69-0.8 (grey zone) is context dependent and a value below 0.69 (green zone) indicates flexible conformation. (**A**) class I human HEB [Q99081], (**B**) class II human MYOD [P15172], (**C**) class III human MYC [P01106-2] and (**D**) *Arabidopsis thaliana* MYC2 [Q39204].

**Figure 2:**
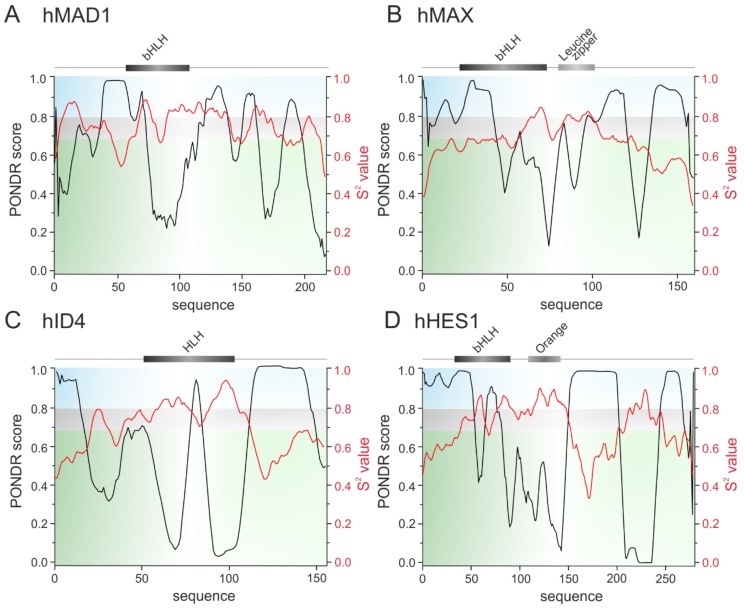
Prediction of intrinsically disordered regions. The top panel presents the domain structure of the analyzed bHLH proteins. Dark grey rectangle indicates the bHLH domain, light grey indicates Leucine zipper or Orange domain. The bottom panel presents a prediction of intrinsically disordered and flexible regions, based on the amino acid sequence of proteins. Predictions were performed using PONDR-VLXT (left Y axis) and DynaMine (right Y axis) software. For PONDR prediction, a score above 0.5 indicates disorder. For Dynamine, a S^2^ value above 0.8 (blue zone) indicates rigid conformation, 0.69–0.8 (grey zone) is context dependent and a value below 0.69 (green zone) indicates flexible conformation. (**A**) class IV human MAD [Q9Y6D9] and (**B**) human MAX [P61244], (**C**) class V human ID4 [P47928], (**D**) class VI human HES1 [Q14469].

**Figure 3:**
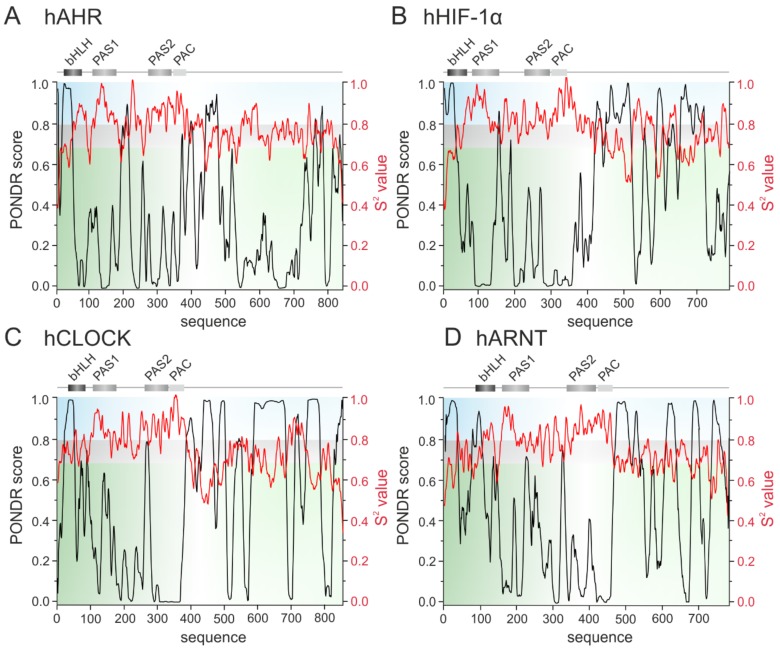
Prediction of intrinsically disordered regions of the class VII bHLH-PAS proteins. The top panel presents the domain structure of the analyzed bHLH–PAS proteins. Dark grey rectangle indicates the bHLH domain, light grey indicates PAS/PAC domains. The bottom panel presents a prediction of intrinsically disordered and flexible regions based on the amino acid sequence of proteins. Prediction were performed using PONDR-VLXT (left Y axis) and DynaMine (right Y axis) software. For PONDR prediction, score above 0.5 indicate disorder. For Dynamine, a S^2^ value above 0.8 (blue zone) indicates rigid conformation, 0.69–0.8 (grey zone) is context dependent and a value below 0.69 (green zone) indicates flexible conformation. (**A**) human AHR [P35869], (**B**) human HIF-1α [Q16665], (**C**) human CLOCK [O08785], (**D**) human ARNT [P27540].

bHLH TFs are responsible for a control of developmental processes like retinal development, proliferation of progenitors, neurogenesis and gliogenesis. Importantly, this is due to a direct interaction between bHLH TFs and interaction of bHLH TFs with homeodomain factors which create complexes that bind to the specific promoters [90,91]. Transcription of muscle-specific genes during skeletal muscle development is also dependent on the interactions between specific bHLH TFs: MyoD, Myogenin, Myf5 and MRF4 with ubiquitously expressed bHLH E-proteins (E12, E47, TCF4, HEB). Interestingly, it was shown that MyoD interacts with two isoforms of HEB: HEBα and HEBβ. which regulate differentially transcriptional activity of MyoD not only on different, but also on the same promoter [92]. Also interesting is the ability of ID4 to recruit multiple ID proteins to assemble higher order complexes. ID4 restores DNA binding by E47 protein even in the presence of repressing ID1 and ID2. Additionally, the ID proteins can interact with non-bHLH partners expanding regulatory network of ID4 [42]. As a consequence, the ID proteins are proposed as a ‘hub’ for coordination of multiple cancer events [27]. These examples illustrate the possibility of bHLH TFs to interact with many partners in differentiated way. We suggest that these is related to the disordered character of the bHLH proteins. This hypothesis is substantiated by some experimental studies. Neurogenic bHLH transcriprion factor Neurogenin 2 (Ngn2) was shown to possess long IDR which phosphorylation regulates the activity of the protein [93]. Interestingly, though the bHLH domain was considered as a stable, well ordered structure, partially disordered character of this domain was presented for NeuroD [94], MYC and MAX [95]. We performed in silico analyses to predict the presence of intrinsic disorder and get an insight into the degree of flexibility of bHLH proteins representing all established classes (see Table 1): hHEB (class I), hMYOD (class II), hMYC and atMYC2 (class III) (Figure 1); hMAD1 and hMAX (class IV), hID4 (class V), hHES (class VI) (Figure 2); hAHR, hHIF-1α, hCLOCK and hARNT (class VII) (Figure 3). We used PONDR-VLXT [96,97], http://www.pondr.com/ for the disorder prediction and DynaMine [98,99], http://dynamine.ibsquare.be/submission/ for prediction of the flexibility of proteins backbone.

A representative of the class I, human HEB shows a high content of predicted as disordered and flexible sequences. The only highly ordered/rigid region appears between 577–630 aa which comprise the bHLH domain (Figure 1A). Based on prediction results, we assume HEB as IDP. Also hMyoD, the class II TFs presents a high content of flexible IDRs especially in the C-terminal part of the protein (Figure 1B). As the representatives of the class III we have chosen hMYC (Figure 1C) (for which partial disorder of the bHLH domain was experimentally documented [95]) and *Arabidopsis thaliana* MYC2 (Figure 1D). For both proteins the presence of flexible IDRs was predicted, though they locations were different.

The representative of the class IV, human MAD1 also shows high content of predicted as disordered and flexible sequences (Figure 2A). Interestingly IDRs of hMAX which belongs to the same class IV are located in the N- and C- protein termini, while the middle part is predicted as possessing more rigid structure (Figure 2B). Also, ID4 belonging to the class V of transcriptional inhibitors presents flexible IDR in the C-terminal part of protein and a shorter one in the N-terminal part (Figure 2C). In addition to similarly located the N- and C-terminal IDRs in the class VI member, human HES1 analysis shows high flexibility/disorder in the central part of protein (Figure 2D).

The class VII proteins comprise the bHLH-PAS subfamily, which additionally to the bHLH domain possess a PAS domain responsible for ligands and co-factors binding. Importantly, their C-termini are usually responsible for the regulation of the protein and created complexes activity [100]. Human AHR, HIF1-α, and CLOCK belong to the subclass I of specialized factors, while human ARNT (the subclass II) is one of the general partners which dimerize with the subclass I proteins and is important for their activity. In contrast to the hAHR, for which relatively short IDRs were predicted within the middle, the N- and the C-terminal part of the protein (Figure 3A), other bHLH-PAS members contain longer IDRs which comprise most of the C-terminal half of proteins and are predicted as highly flexible (hHIF-1α, Figure 3B; hCLOCK, Figure 3C; hARNT, Figure 3D).

To date, the only report, concerning the structure of the full-length bHLH protein is the mentioned study showing Neurogenin as IDP [93]. Based on the presented predictions and our own experience with expression of the selected bHLH proteins (not published), we assume that this is due to the relatively high content of IDRs. This makes overexpression and purification process extremely difficult because of propensity to aggregation and high sensitivity to proteases.

## 4. The Role of IDPs in Maintaining/Creation of LLPS

Over the last decade, since the pioneering work regarding physical nature of P-bodies was published by Hyman and co-workers [101], many molecular biologists and biophysicists have focused on the significance of spontaneous thermodynamically driven liquid-liquid phase separation (LLPS) in biological systems. LLPS leads to formation of dense, liquid condensates that stably coexist in diluted phase [101,102]. At the molecular level it was shown that LLPS is forced by multiple weak and transient interactions which engage IDPs/IDRs [101,103,104,105,106]. Repetitively distributed within IDRs highly charged regions of opposite charges, short motifs such as YG/S-, FG-, RG-, GY-, KSPEA-, SY- and Q/N-rich regions form multivalent interactions between condensate components [107]. A model for the condensate formation and composition proposes that some proteins act as the scaffolds, while others as the clients. The scaffolds are the modular proteins which contain repeated motives that enable heterotypical scaffold-scaffold interaction. As they undergo spontaneous LLPS they are essential for the structural integrity of a condensate [108,109]. Directly interacting sequences called stickers are usually multivalent, whereas the interval sequences which separate stickers, called spacers are responsible for the properties of a condensate [110]. Highly charged and flexible IDRs are in fact frequently identified as scaffolds [108,111]. The clients participate into the condensates by binding to the free, unoccupied scaffold sites [108]. A growing number of evidences indicate that LLPS constitute a fundamental mechanism to compartmentalize the intracellular space. LLPS form the functional centres for biochemical reactions in cytoplasm and membrane-surrounded organelles including nucleus.

The structural and functional organisation of the interior of the nucleus was believed to rely solely on the rigid insoluble nuclear matrix [112]. The rich in A and T DNA sequences known as scaffold/matrix associated regions (S/MARs) attach to nuclear matrix and organise chromatin into higher-order structures which comprise distinct loops and functional units attached to the matrix [113]. That concept is now giving way to a new concept, were dynamic, spontaneously formed condensates, such as nucleolus, splicing speckles, Cajal bodies, PML bodies are the key structural and functional components of the nuclear interior. The barrier-free character of liquid condensates allows for rapid exchange of their components with surrounding so they form an ideal environment for biochemical reactions. On the other hand, nuclear condensates have a stable inert, well-defined structure and can be purified by biochemical methods [114]. It was shown, that the concentration of nucleolar components is close to saturation [115]. It means that small changes in the nucleus can drive spontaneous LLPS. In fact association/dissociation events of nuclear condensates regulate many processes related to gene expression [116] including chromatin structure organisation [117], RNA processing [118], ribosome biogenesis [119]. Importantly, LLPS was shown to be involved in formation of some functional condensates that regulate genes transcription [76,120,121,122].

## 5. The Transcription Regulation and LLPS

The genes transcription process require tight regulation to ensure physiological balance of the cell. Knowledge regarding the mechanism of transcription is quite advanced, however some aspects of regulation remains unexplored. Recent findings indicate that regulatory mechanism may tightly depends on the spontaneous LLPS. Transcription of tissue specific gene is initiated at the specific genome regions called super-enhancers (SE). SE first described in embryonic stem cells (ESC) [123] are dense multicomponent assemblies different from typical enhancers [124]. Recently Hnisz [125] performed computational simulation to obtain the probable explanation for typical features of SE. Simulations led to conclusion that formation, activity and unique properties of SE such as sensitivity to concentration of its components, sensitivity to posttranslational modifications, extremely high frequency bursting [126,127,128] may originate from the fact that SE are liquid condensates assembled/disassembled via spontaneous LLPS [125]. Hnisz and co-workers were the first who point connection and strong dependence between the regulation of transcription initiation at SE and LLPS. Although not experimentally proven, the model serves as the conceptual framework for further research. Recently, Sabari et al. [121] showed that largely disordered BRD4 and MED1 subunit of the Mediator are in close spatial proximity to one another within SE in murine ESC and co-localised puncta show characteristic features of phase separated condensates Moreover, MED1 condensates can incorporate BRD4 and Pol II from nuclear extract [121]. MED1 subunit interacts also with other major pluripotency TFs e.g., OCT-4 [129] and estrogen receptor (ER) [130] forming liquid-like puncta at SE of the key pluripotency genes [121,122]. MED1 condensates depends on the OCT-4 occupancy [122], which are crucial for initiation of tissue specific genes transcription at SE [122,131]. In vitro analyses pointed that formation of MED1-OCT4 liquid condensates occurs via the electrostatic interactions and involves acidic residues enriched in disordered activation domain of the OCT-4 [122]. Interestingly, ER interact with the MED1 subunit by LXXLL motif [132] which is located in the ordered ligand binding domain. This interaction is regulated by estrogen what means that not only disordered-disordered regions interaction but also disordered-ordered regions interactions play a role in transcription regulation forced by LLPS [122]. Wu et al. [120] showed that largely disordered transcription co-activator TAZ protein forms liquid condensates in vitro and *in vivo*. TAZ condensates compartmentalize DNA binding cofactor TEAD4 and other components of transcription initiation machinery including BRD4, MED1 and CDK9. Importantly, deletion mutant, that is not able to undergo spontaneous LLPS cannot initiate transcription though is able to bind TAZ partners such TEAD4.

Importantly, there are some evidences that not only the initiation, but also the elongation of transcription depends on LLPS. For the transcription elongation essential is hyper-phosphorylation of the YSPTSPS consensus sequence which is repeated multiple times in the disordered C-terminal domain (CTD) of Pol II [133,134,135,136]. pTEFb which begins the elongation phase consists of CDK9 kinase associated with cyclin T1 (CycT1). Lu with co-workers [76] concentrated on the function of the lengthy C-terminal IDR of CycT1 in regulation of CDK9 activity. They revealed that a histidine-rich domain (HRD) located in the IDR of CycT1 (residues 480–550) is directly involved in the regulation of the kinase activity [76]. Interestingly, HRD is present also in some other kinases, for example Dyrk1A which phosphorylates CTD of Pol II. Importantly, a homologues kinase Dyrk3 was shown to be responsible for disassembly of stress granules [137] and other cellular condensates during cell division [138]. In vitro studies using a set of recombinant IDRs of the CycT1 and Dyrk1A revealed that the regions can undergo phase separation in a HRD dependent manner. HRD was shown to form condensates which compartmentalize the kinases and the substrate what enables efficient reactions resulting in the hyper-phosphorylation of the CTD of Pol II [76]. Interestingly, the CTD of Pol II can undergo spontaneous LLPS in vitro only in a non-phosphorylated state. The weak CTD-CTD interaction keeps the enzymes molecules in hubs within nucleoplasm. Phosphorylation change the interaction pattern allowing CTD to engage in new multivalent interactions with selected partners [139]. These results indicate that LLPS allows for the condensation of cofactors, that in turn triggers posttranslational modifications leading to the reorganization of the condensate components. Pol II escapes from the promoter site and enables the entry into active elongation stage [76].

**Figure 4:**
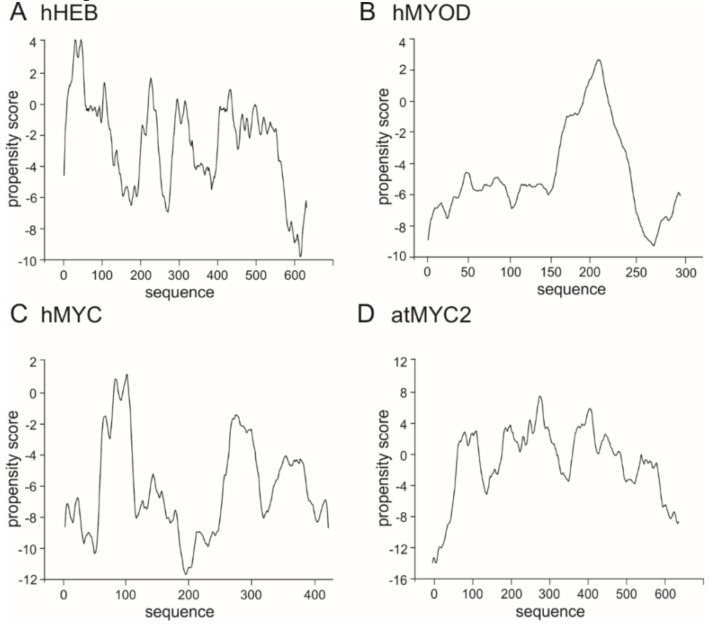
Prediction of propensity of LLPS formation. (**A**) class I human HEB [Q99081], (**B**) class II human MYOD [P15172], (**C**) class III human MYC [P01106-2] and (**D**) *Arabidopsis thaliana* MYC2 [Q39204].

**Figure 5:**
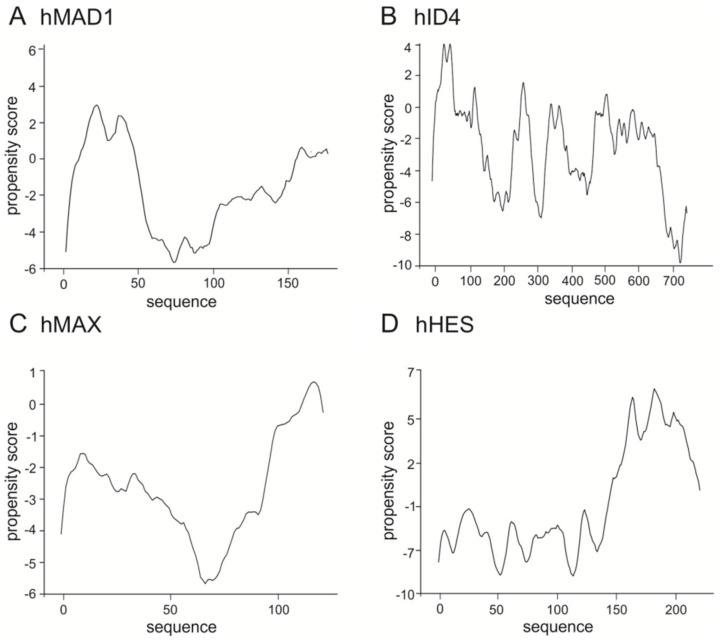
Prediction of propensity of LLPS formation. (**A**) class IV human MAD [Q05195] and (**B**) human MAX [P61244], (**C**) class V human ID4 [P47928], (**D**) class VI human HES1 [Q14469].

**Figure 6:**
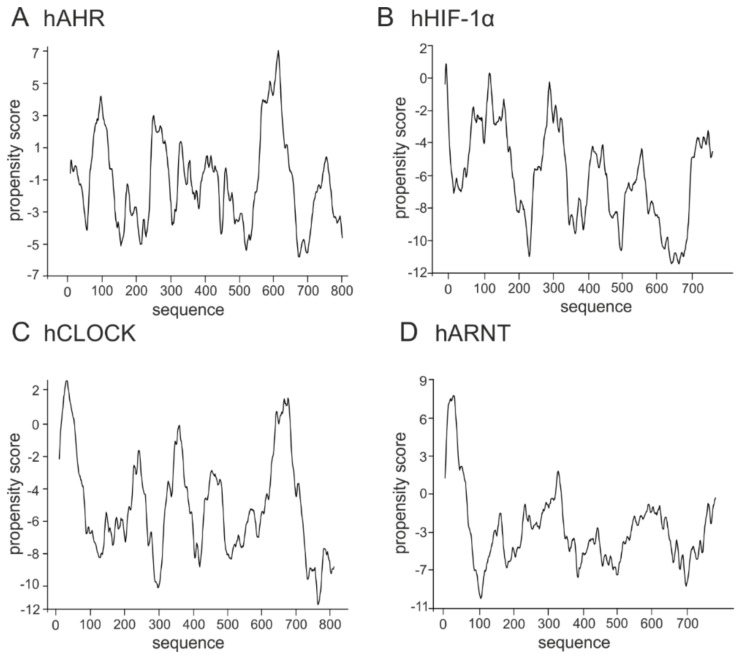
Prediction of propensity of LLPS formation for bHLh-PAS proteins. (**A**) human AHR [P35869], (**B**) human HIF-1α [Q16665], (**C**) human CLOCK [O08785], (**D**) human ARNT [P27540].

Currently not much is known about proteins responsible for formation of the condensates which are important for transcription regulation. The question still remains unanswered which proteins are the scaffolds and which are the clients. Importantly, also not much is known about the involvement of the bHLH TFs in the LLPS process, though they are key players involved in many important cell differentiation and organisms development pathways. As we discussed in previous section, bHLH proteins possess long IDRs which could interact with different partners and be engaged in LLPS. This hypothesis is substantiated by an experimental verification of MyoD possibility to create LLPS [122], and discussed in previous section possibility of some bHLH TFs to interact with the Mediator subunits or other elements of the mechanism which modifies the chromatin accessibility. Interestingly, regulation of circadian clock by BMAL1 comprises binding of CBP, which occurs in discrete nuclear foci. This led to a hypothesis that formation of nuclear bodies containing BMAL1/CBP provides transcriptionally active sites of target genes, like *Per1-2* [34]. Taking the above into consideration, we asked the question if the ability to undergo LLPS is a more general property of the bHLH TFs. As we got positive results for the previously performed prediction of disorder, which was shown to be important for LLPS initiation [76,121,122], we decided to perform in silico analyses to predict if members of the bHLH family comprise putative sequences able to create liquid condensates. We used catGranule program, (http://service.tartaglialab.com/update_submission/216885/dd56e32a89) for computational analyses of the putative propensity to undergo LLPS [140] for the bHLH proteins representing all established classes (see Table 1). Prediction results showed that hHEB (class I), hMyoD (class II), hMYC and 84atMYC2 (class III) (Figure 4) contain sequences with a positive score of propensity to LLPS formation. Interestingly, proteins from the class IV regulators which do not possess TAD: hMAD1 and hMAX, similarly like transcription repressors: hID4 (class V) and hHES (class VI) present very low or even negative score within the whole protein sequence (Figure 5). bHLH-PAS transcription factors representing the class VII, hAHR, hHIF-1α, hCLOCK and hARNT were predicted as containing some sequences with high propensity score (Figure 6). Especially interesting is the observation that the transcription repressors show a very low propensity scoreto undergo LLPS in contrast to the transcription activators such as hHEB or atMYC2. It is possible that the bHLH repressors inhibit transcription by preventing spontaneous phase separation required to form a complete initiation complex. This hypothesis is substantiated by the observation for TAZ mutants [120], discussed in the previous section.

**Figure 7:**
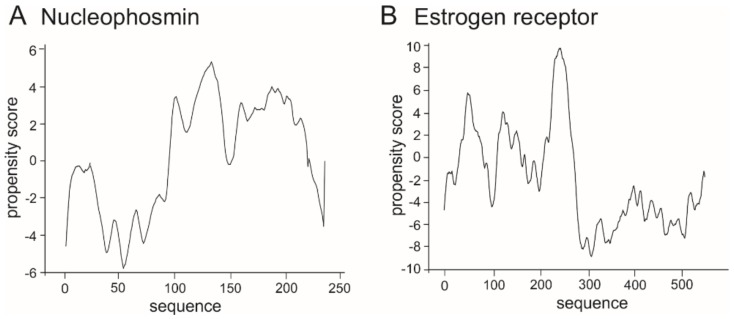
Prediction of propensity of LLPS formation for representative LLPS-enabled proteins. (**A**) nucleophosmin [P06748], (**B**) estrogen receptor [P03372].

As the range of the propensity score is not determined precisely, as a control we performed catGranule prediction for proteins known to create LLPS: nucleophosmin (Figure 7A) and estrogen receptor (Figure 7B) which are deposited in the recently published PhaSePro database (https://phasepro.elte.hu) [141].

Results of performed in silico analyses in comparison to the control show that the selected bHLH proteins have regions that might be involved in multivalent interaction leading to formation of liquid condensates. What would be their role in condensates formation and how would mutations and wrong dimerization/interaction influence formation of the bHLH TFs containing condensate remains a puzzle, however we believe that such an important family of TFs engaged in the crucial pathways and related to many severe disorders like cancer should be the subject of research in this field.

## 6. Concluding Remarks and Future Perspectives

In eukaryotic cells, regulation of transcription is a dynamic process which requires very precise temporal and spatial coordination of proteins assembling functional complexes. The bHLH family comprises a large group of TFs which utilize conserved DNA binding domain to interact with DNA, but also additional, often disordered domains and motives that allows formation of complex interacting network with various transcription co-factors. It is possible that flexible disordered regions of the bHLH proteins play a role in formation of liquid condensates via LLPS and contribute in this way to regulation of transcription process. Up to date however, there is a lack of experimental evidences. Also recently published PhaSePro database for LLPS does not contain any bHLH TF [141]. We believe that this is due to difficulties with the experimental studies of the bHLH proteins mentioned previously and we expect that some bHLH proteins will be appended in future.

Presented in the previous section predictions may give a hint about the link between LLPS by the bHLH proteins and transcription regulation. This raise a question about functional relevance of this discrepancy between family members. An interesting observation is the predicted low propensity score to form LLPS in the case of transcriptional repressors in contrast to proteins acting as activators. This raise a question about the functional relevance of this discrepancy between family members. Importantly, connection between LLPS and transcription regulation is not limited to the direct interaction between transcription regulators at the active transcription sites. LLPS form nuclear bodies, that maintain, store and modify transcription regulators. Examples include nuclear speckles, polyleukemia bodies, nucleolus, histone locus and others [142]. Within LLPS-formed condensates proteins can undergo acetylation/deacetylation or sumoylation, proteasome-dependent degradation and other posttranslational modifications that influence their functionality [143,144,145]. Importantly, barrier-free character of these phase separated condensates allows shuttling of its component between the condensates and nucleoplasm, and whenever needed molecules can be recruited from these compartments to the active transcriptionally sites. The discovery that LLPS which is well known in polymer chemistry can play an important role in molecular biology has definitely brought us closer to understanding the cell functionality and regulation of fundamental cellular processes such as transcription. However, our understanding and detailed knowledge is still residual. Many important questions regarding a LLPS concept in transcription regulation remain without answer. We do not know, which components drive association/dissociation events at the active sites. Which molecules serves as a scaffold conditioning formation of liquid condensates and which are just clients. How the type of client molecules influence the function of the phase separated condensates? Also, we do not know which factors and in which way alter LLPS leading to the pathological processes. What would be the role of the bHLH TFs in a condensates formation, and how mutations and incorrect dimerization/interaction of these proteins would impact formation and function of condensates? These questions, as well as many other ones await experimental verification. We believe that such important family of transcription factors which is engaged in crucial pathways and related to many severe diseases like cancer and neurodegenerative disorders, should be the subject of further intensive studies.

## Figures and Tables

**Table 1:** Classification of bHLH proteins based on [5,7,8,10,12,14,16].

Structural Motif Dimerization	Representative Members	Short Description
**class I (E proteins)/ group A**		
bHLH, homo- and heterodimerization	Vertebrate: E12, E47 [17], HEB [18,19], TCF4 [20] Invertebrate: Daughterless	transcription activators, ubiquitous expression, neurogenesis, immune cell development, sex development, gonadogenesis
**class II/ group A**		
bHLH, preferred heterodimerization with class I partners	Vertebrate: MYOD, Myogenin, MYF5-6, Ngn1-3, ATOH, NeuroD, NDRF, MATH, MASH, ASCL1 [21], TAL1/SCL [22], OLIG1-3 [23] Invertebrate: TWIST [24], AS-C	transcription activators, tissue specific expression, muscle development, neuro-genesis, generation of autonomic and olfactory neurons, development of granule neurons and external germinal layer of cerebellum, oligodendrocyte development, specification of blood lineage and maturation of several hematopoietic cells, pancreatic development
**class III/ group B**		
bHLH-LZ	Vertebrate: MYC [25], USF, TFE3, SREBP1-2 *Drosophila*: MYC Plants: MYC2	transcription activators/represors, oncogenic transformation, apoptosis, cellular differentiation, proliferation, cholesterol-mediated induction of the low-density lipoprotein receptor, jasmonate signaling (plants)
**class IV/ group B**		
bHLH, heterodimerisation with each other and MYC proteins	Vertabrate: MAD, MAX [26], MXI1 *Drosophila*: MNT, MAX	transcription regulators lacking transactivation domain (TAD)
**class V/ group D**		
HLH (no basic region)	Vertebrate: ID1-4 [27] Invertebrate:EMC	negative transcription regulators of class I and II (group A) proteins, no DNA binding, regulation by sequestration.
**class VI/ group B**		
bHLH-O, (presence of proline in basic region)	Vertebrate: HES, HEY1-3 [28], STRA13, HERP1-2 [29] *Drosophila*: HAIRY [30], E(spI)	negative transcription regulators interacting with corepressors (Groucho); neurogenesis, vasculogenesis, mesoderm segmentation, myogenesis, T lymphocyte development, cardiovascular development and homeostasis; effectors of Notch signalling [28]; in *Drosophila*: regulation of differentiation, anteroposterior segmentation and sex determination
**class VII/ group C - subclass I**		
bHLH-PAS, heterodimerization with subclass II	Vertebrate: AHR [31], HIF1-3α [32], SIM1-2 [33], CLOCK [34], NPAS1-4 [35,36,37,38,39] *Drosophila*: MET [40], GCE, SIMA, TRH	transcription regulation in response to physiological and environmental signals: xenobiotics, hypoxia, development, circadian rhytms
**class VII/ group C - subclass II**		
bHLH-PAS, homo- and heterodimerization with subclass I	Vertebrate: ARNT [41], ARNT2, BMAL1, BMAL2 *Drosophila*: TANGO, CYCLE	general partners for subclass I bHLH-PAS proteins

## Abbreviations

AHR	Aryl hydrocarbon receptor
AS-C	Achaete scute complex
ARNT	Aryl hydrocarbon receptor nuclear translocator
bHLH	Helix–loop–helix
ccRCC	Clear cell renal cell carcinoma
CLOCK	Circadian locomotor output cycles protein kaput
CTD	C-terminal domain
CycT1	Cyclin T1
EMC	Extramacrochaetae
E(spI)	Enhancer of split
ER	Estrogen receptor
ESC	Embryonic stem cells
GCE	Germ cell-expressed protein
GRO	Groucho
HAT	Histone transacetylase
HIF	Hypoxia-inducible factor
HSCa	Hematopoietic stem cells
HRD	histidine reach domain
ID	Inhibitor of DNA binding
IDPs	Intrinsically disordered proteins
IDRs	Intrinsically disordered regions
LLPS	liquid-liquid phase separation
LZ	Leucine zipper motif
MET	Methoprene-tolerant protein
MXI1	Max interacting protein
Ngn2	Neurogenin
NPAS	Neuronal PAS domain-containing protein
PAS	Period-arylhydrocarbon nuclear translocator-single minded domain
Pol II	RNA polymerase II
SE	Super-enhancer
SIM	Single-minded protein
SIMA	Similar protein
S/MARs	Scaffold/matrix associate regions
SREBP	Sterol-responsive element-binding protein
TAD	Transactivation domain
TAZ	Tafazzin
TFs	Transcription factors
TRH	Trachealess protein
USF	Upstream stimulatory factor
VHL	Von Hippel-Lindau tumor suppressor
